# Cover

**DOI:** 10.4269/ajtmh.1114_scover

**Published:** 2024-10-01

## Abstract

The cover artwork shows a lymphedema patient and caregivers undertaking lower-limb washing. It emphasizes the important role caregivers and trainers provide in ensuring that regular hygiene measures are followed for patients with lymphedema. We are grateful to Lauren Horton, the artist, who produced the draft images based on material publicly available and then developed the final work with input from several authors of this volume.

*Photo Credit*: Lauren Horton, original artwork, 2024

